# A Comparative Study of Helical and Cross-Wedge Rolling Processes for Producing Ball Studs

**DOI:** 10.3390/ma12182887

**Published:** 2019-09-06

**Authors:** Tomasz Bulzak, Janusz Tomczak, Zbigniew Pater, Krzysztof Majerski

**Affiliations:** Faculty of Mechanical Engineering, Lublin University of Technology, 36 Nadbystrzycka Str., 20-618 Lublin, Poland (J.T.) (Z.P.) (K.M.)

**Keywords:** rolling, cross-wedge rolling, helical-wedge rolling

## Abstract

This paper presents two rolling technologies: cross-wedge rolling (CWR) and helical-wedge rolling (HWR). The two rolling processes were compared using the example of rolling a ball stud forging. The technologies were modeled in the finite element model (FEM) environment. Calculations were performed to obtain distributions of strain and the Cockcroft–Latham damage criterion. The investigated processes were also performed under real-life conditions. The results of the experiments were used to compare the force and energy parameters of the rolling technologies. Tests were also carried out to investigate the microstructure of the studs and a grain size after rolling. The state of the macrostructure, i.e., the grain flow lines, was also compared. The experiments showed that HWR was a more energy-efficient process.

## 1. Introduction

Cross-wedge rolling (CWR) is a technology used mainly to form axisymmetrical products [1]. Tools with a polygonal cycloid profile, however, provide the possibility to form products with non-axisymmetric (e.g., square, triangular, oval) cross-sections from circular cross-section billets [2]. Cycloid-shaped wedge tools can also be used for rolling camshafts for an internal combustion (IC) engine [3]. CWR is used in the manufacture of grinding media for ball mills [4]. This technology is also employed in the production of rail screw spikes [5]. Cross-rolling of external threads is a technology that shares many common features with CWR, but is classified as a separate group of thread rolling processes [6].

CWR can be performed according to many kinematic variants [7]. Most often, however, rolling mills use a system of two rolls [8]. Less common are rolling systems using flat tools [9]. Rolling with two rolls is more efficient, but requires the use of guides to hold the workpiece in the space between the rolls. Two-roll mills have the additional advantage of occupying less space than flat-wedge mills. A flat-wedge reversing rolling mill has also been constructed, in which lost motion of the slide was eliminated. The mill, which uses two sets of tools (an upper and a lower tool set), allows one to roll, in one duty cycle (the advance and return of the hydraulic cylinder), one product in two operations or two products, each requiring one operation [10]. In the literature, one can also find theoretical considerations concerning cross-wedge rolling of hollow products in a three-roll system [11].

A wedge for cross-wedge metal forming can be helically wound on the roll face [12]. The process in which such tools are used is called helical-wedge rolling (HWR). This solution allows one to shape axisymmetric products in a continuous manner from one section of a billet (round bar). The process is characterized by very high efficiency. HWR is mainly applied in the production of steel balls [13], but there are also reports on the use of this process in the production of preforms and studs [14]. Despite the many advantages offered by HWR, CWR is the more commonly used process. Perhaps HWR is too efficient, which is why its application in the manufacture of products other than steel balls is unprofitable. Grinding media balls, after the rolling process, are only subjected to hardening, which does not reduce the efficiency of the entire process. In order to exploit the production efficiency potential of HWR, it is necessary to use an efficient induction heating system, which, however, requires high voltage power supplies, and is associated with high costs. Another limitation of HWR is that it is difficult to achieve a high efficiency of finishing of rolled products. Yet another problem is the designing of tools, which is not a simple process [15].

**Figure 1:**
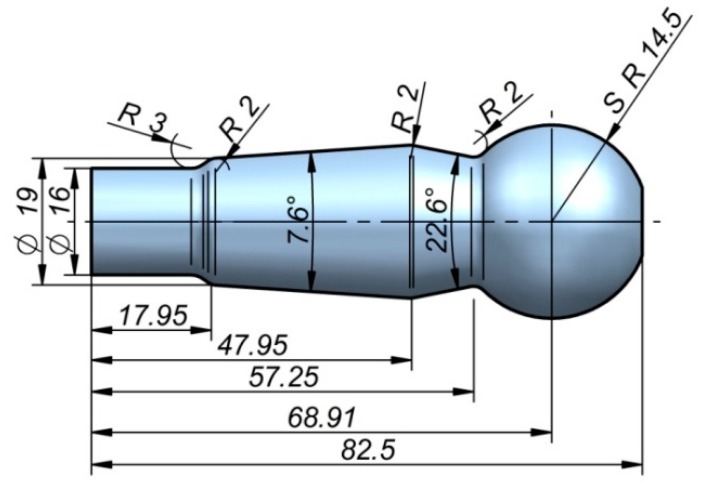
Ball stud used in the analysis of cross-wedge rolling (CWR) and helical-wedge rolling (HWR), with the dimension in mm given.

CWR and HWR technologies have quite similar technological possibilities. The differences result from a different kinematic of the process realization, as well as tool design. In order to compare these two technologies in terms of force parameters and product quality, a comparative analysis was conducted. The research published so far has concentrated mostly on the CWR technology. There is significantly less information on the HWR process. The performed research aims to present the differences and similarities of these two technologies. This paper presents a comparison of CWR and HWR, using the example of rolling the ball stud shown in Figure 1.

## 2. Parameters of CWR and HWR Processes

**Figure 2:**
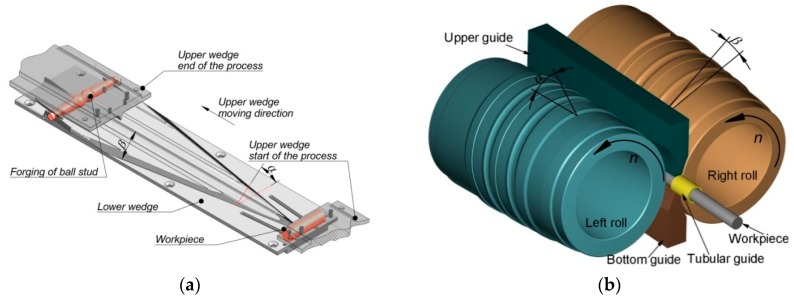
Schematics of: (**a**) cross-wedge rolling (CWR); (**b**) helical-wedge rolling (HWR).

CWR was carried out in a rolling mill [16] consisting of two flat tools (Figure 2a). The geometry of the tools was described by the following parameters: forming angle *α* = 30° and spreading angle *β* = 10°. The upper wedge tool moved at a speed of 300 mm/s. The billets were ∅ 28 mm × 110 mm C45 steel round bars. HWR was performed using cylindrical tools with a maximum diameter ∅ 300 mm (Figure 2b) [17]. The rotational speed of the rolls was 30 rpm. The geometry of the cylindrical tool was described by the following parameters: forming angle *α* = 30° and spreading angle *β* = 5°. The billets processed by HWR were ∅ 28 mm × 350 mm C45 steel round bars. The chemical composition of the C45 steel used in the tests is presented in Table 1. CWR was simulated with Simufact Forming 15.0 software, and the billet was discretized using hexagonal elements with an average size of 1 mm. Numerical simulations of HWR were performed with Forge NxT 1.1 software, and the billet was discretized using tetragonal elements with an average size of 1 mm. In the analysis, two types of finite element modeling (FEM) software were used. Firstly, the analysis focused on the CWR process, for which we used the Simufact Forming 15.0 program, since it met our requirements regarding the simulation of the CWR process. In the case of the HWR, Simufact Forming 15.0 had difficulties simulating the slitting of studs during the process. For this reason, we applied Forge NxT 1.1 software, which is much better at separating material (it does not remove the lower-volume part). Due to this fact, Forge NxT 1.1 was used in further parts of the study. In both cases, however, the same material model and boundary conditions were applied. This is also why the simulation of the CWR process was not repeated in Forge NxT 1.1. Prior to rolling (both CWR and HWR), the billet material was heated to 1100 °C. The temperature of the rolls and the flat tools during the process was 50 °C. Thermal contact between the workpiece and the tools was described by a heat transfer coefficient of 10 kW/m^2^K. Mechanical contact was modeled using the Tresca friction model, for which the friction factor was 0.9. For the HWR process, the critical value of the trigger characteristic was used to initiate the deletion of finite elements, which allows for the division of the batch material. The critical trigger value was estimated based on the Cockcroft–Latham criterion. It was assumed that the trigger’s critical value is 2.75. Thermal parameters of C45 grade steel, such as thermal conductivity (700 °C—30.1 W/mK; 800 °C—24.7 W/mK; 900 °C—28.2 W/mK; 1000 °C—30.8 W/mK; 1100 °C—26.7 W/mK; 1200 °C—27.1 W/mK), specific heat (700 °C—6.1 kJ/kgK; 800 °C—6.5 kJ/kgK; 900 °C—7.3 kJ/kgK; 1000 °C—12.8 kJ/kgK; 1100 °C—7.8 kJ/kgK; 1200 °C—6.5 kJ/kgK), and emissivity (0.7) were considered. Within the numerical model, it was assumed that the value of flow stress $\sigma_{p}$ depends on strain *ε*, strain rate $\dot{\varepsilon }$, and temperature *T*. For the calculations, the Huber–Mises plastic criterion was employed. The workpiece material was modeled as a rigid-plastic object. The workpiece was modeled as isotropic material. The rheology of C45 steel in Simufact Forming 15.0 and Forge NxT 1.1 was described by the Hensel–Spittel Equation (1):

(1) $$\sigma_{p}=1521.31\times e^{-0.0027T}\times \varepsilon^{-0.1265}\times e^{-0.05958/\varepsilon }\times {\dot{\varepsilon }}^{0.1454},$$

where: *σ_p_*—yield stress (MPa), *ε*—effective strain (*ε* = 0.05–1.5), *T*—temperature (*T* = 700–1250 °C), and —strain rate ($\dot{\varepsilon \;}$= 0.01–500 s^−1^).

**Figure 3:**
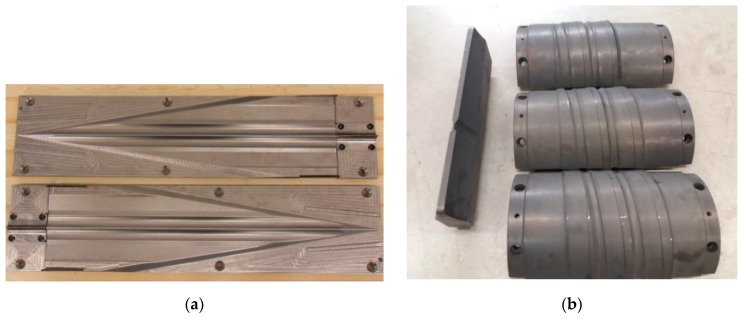
Tools used in: (**a**) cross-wedge rolling (CWR); (**b**) helical-wedge rolling (HWR).

The tool sets used in the experiments are shown in Figure 3. The set of tools used in CWR included two plates. The tools used in HWR were two rolls and two guides. The rolls used in the study had a segmented structure, i.e., each roll consisted of three rings with a central angle of 120°. The tools had been manufactured by the same producer, according to the same standards. The cost of the tools used in HWR was more than double that of the flat CWR tools. The experimental tests were carried out under the conditions defined for the numerical calculations.

## 3. Results

**Figure 4:**
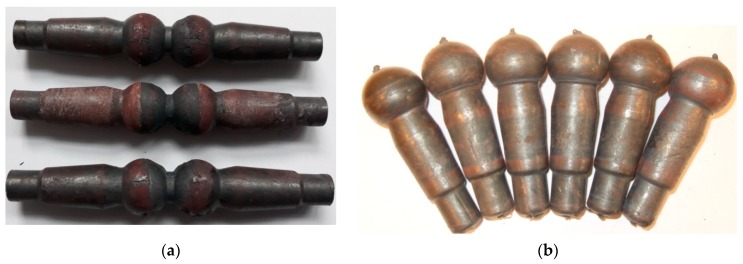
Ball studs obtained by: (**a**) cross-wedge rolling (CWR); (**b**) helical-wedge rolling (HWR).

**Figure 5:**
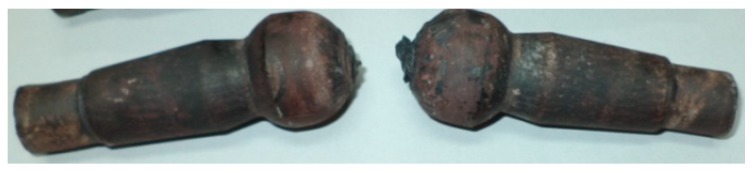
Ball studs after cutting during cross-wedge rolling (CWR).

In the CWR experiments, two studs were formed from a 110-mm-long billet during one duty cycle. In one cycle of HWR, we obtained as many as seven studs from one 350-mm-long billet. The ball-stud forgings formed using the investigated methods are shown in Figure 4. The forgings rolled using the CWR method were connected by a bridge (Figure 4a). It is generally possible to separate the forgings during CWR; however, in the analyzed case, the forgings bent on the cutting knife during the separation. As a result, the spherical part of the stud (the head) was deformed as shown in Figure 5. The studs obtained by HWR were also separated during rolling, but no negative events affecting the quality of the studs were observed. In Figure 4b, one can see small remains of the connectors that held together the studs produced by HWR. In CWR, end waste was present on both produced studs. In HWR, end waste was found on only two of the seven obtained studs. In the case of HWR, the amount of waste per stud can be further reduced by increasing the length of the round bar billet.

**Figure 6:**
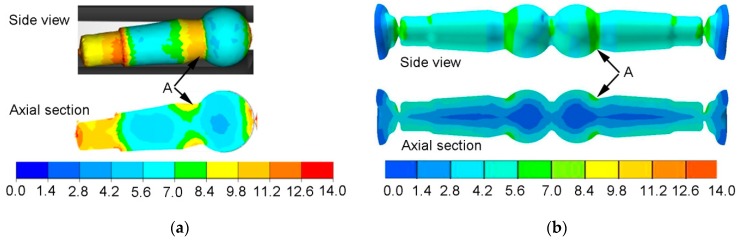
Distribution of effective strain: (**a**) helical-wedge rolling (HWR) ball stud; (**b**) cross-wedge rolling (CWR) ball stud.

Figure 6 shows a comparison of strain rate distributions in studs rolled using HWR and CWR. The nature of the distributions and the values of strain rate were different. Higher strains were recorded for HWR. In both cases, increased strain values were located in area A. For HWR, strains in area A reached a value of over 9.8. In the case of CWR, strains in area A were higher than 5.6 and lower than 7. The elevated strains observed in the studs rolled using HWR may be due to the longer forming path associated with the smaller value of angle *β*. The length of the forming path (length of the wedge) was *L* = 680 mm for CWR, and *L* = 3000 mm for HWR. The distribution of strains in the cross-wedge-rolled stud was layered. The layers with different strain values ran along the axis of the stud. The strain values changed in the radial direction. The highest strains were located in the layers near the surface of the stud, and the lowest along its axis. In the case of the HWR stud, the strain values can be assumed to change in the radial direction, but there was also a large variation in strain in the axial direction. The lowest strains were located in the head of the stud and the longer, tapered part of the shank, while the highest strains occurred in the end step of the stud (the stem), which has the smallest diameter. Similar to the CWR stud, the strains in the layers near the surface were higher than in the central layers. In the case of HWR, higher strains were also observed at the ends of the stud, which is a result of twisting of the material during separation of finished studs.

**Figure 7:**
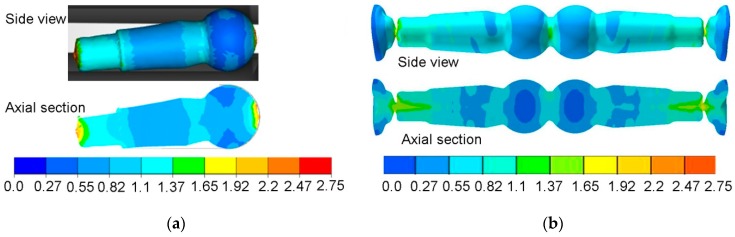
Distribution of the normalized Cockcroft–Latham criterion: (**a**) helical-wedge rolling (HWR) stud; (**b**) cross-wedge rolling (CWR) stud.

Figure 7 shows the distribution of the normalized Cockcroft–Latham damage criterion. The value of the Cockcroft–Latham criterion was determined from Equation (2):

(2) $$CL=\int_{0}^{\varepsilon }\frac{\sigma_{1}}{\sigma_{i}}d\varepsilon$$

where: *CL*—Cockcroft–Latham criterion, $\sigma_{1}$—maximum principal stress, $\sigma_{i}$—effective stress, *ε*—effective strain.

**Figure 8:**
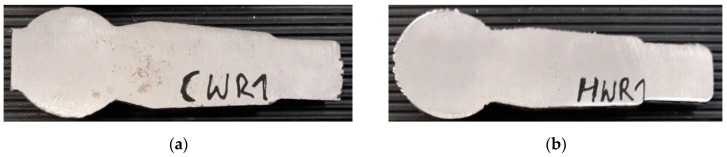
Ball studs obtained by: (**a**) the cross-wedge rolling (CWR) method; (**b**) the helical-wedge rolling (HWR) method, cut along their axis.

In both rolling processes, an increase in the damage criterion was observed only in small areas of the forgings. In the case of the HWR stud, increased values of the *CL* criterion were found at its ends. In these areas, the material had been subjected to controlled cracking in order to separate the neighboring studs. In the remainder of the HWR stud, the *CL* criterion had values below 1, which are safe for C45 steel. In the case of the stud rolled using the cross-wedge method, increased values of the *CL* criterion were observed in the central areas of the stem. The *CL* values for this area were about 1.5. Comparing these two rolling methods, one can conclude that unfavorable stress-strain states can cause cracking to occur in different parts of rolled studs, as shown by the maximum values of the Cockcroft–Latham criterion. In practice, however, these types of processes can lead to so-called low-cycle fatigue of material, known as the Mannesmann effect. To see whether the forgings obtained by CWR and HWR had internal cracks produced by the Mannesmann effect [18], the studs were cut along their axis as shown in Figure 8. No internal cracks were observed in either case.

**Figure 9:**
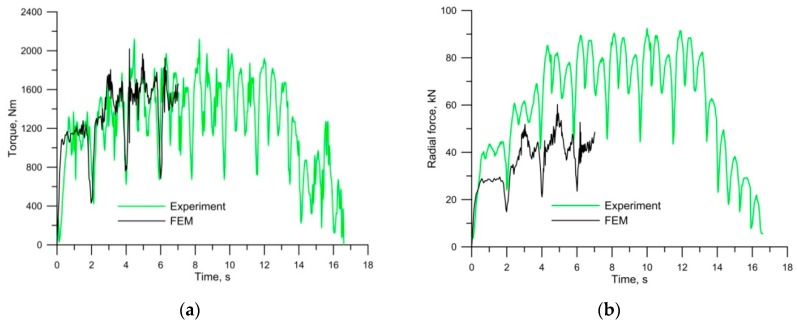
Force distribution parameter for helical-wedge rolling (HWR): (**a**) torque; (**b**) radial force.

Figure 9 shows the force parameters of HWR. Both the torque and the expansion force changed cyclically. The rate of these changes was closely related to the rotational speed of the rolls. When the wedge cut into the material, the torque and the expansion force increased every turn. The torque did not exceed 2000 Nm and the expansion force was not larger than 90 kN. The force parameters for the FEM model were obtained for only the first seven seconds of the process—because rolling is a continuous process, numerical simulations were carried out only until the determined process phase had been reached. Determination coefficient *R*^2^ constitutes the measure of the accuracy of the FEM-based model. The measured and calculated (FEM) curves of torque show very good agreement, both in qualitative and quantitative terms (*R*^2^ = 0.87). In the case of the radial force, the curves are in qualitative agreement, but quantitatively they show discrepancies (*R*^2^ = 0.73). During the FEM calculations, the value of the radial force was underestimated.

**Figure 10:**
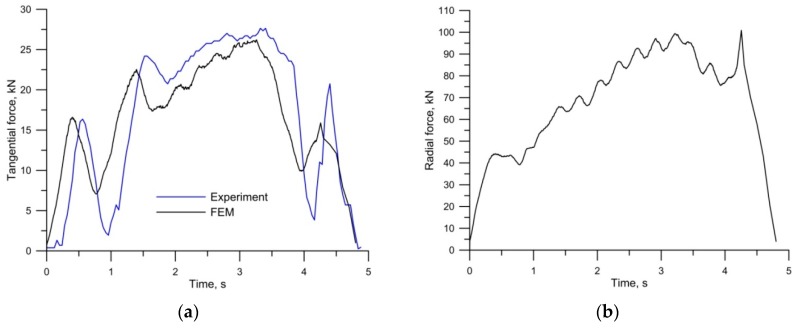
Force distribution parameter for cross-wedge rolling (CWR): (**a**) tangential force; (**b**) radial force.

Figure 10 shows the force parameters of CWR. During the rolling of the studs in the mill with two working tools, the tangential force (pushing the tool) reached the maximum value of 27.5 kN. The experimental and calculated (FEM) force curves were in very good agreement, both qualitatively and quantitatively (*R*^2^ = 0.9). In the case of the radial force, we only have FEM data. The maximum value of the radial force of 100 kN observed in the CWR process is similar to that obtained during HWR.

**Figure 11:**
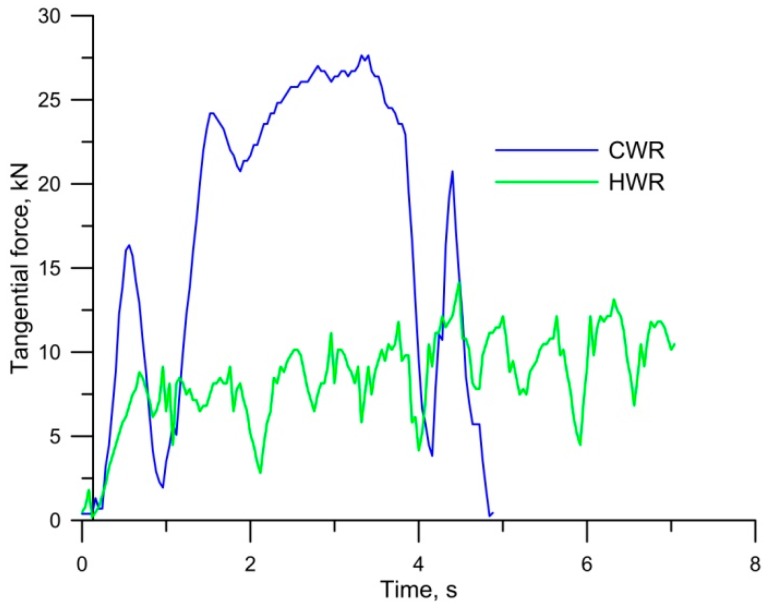
Comparison of tangential force for cross-wedge rolling (CWR) and helical-wedge rolling (HWR).

Figure 11 shows tangential force curves for CWR and HWR. The tangential force for CWR was measured directly during rolling. The tangential force for HWR was determined on the basis of FEM results. The results indicate that the maximum tangential force observed during HWR was twice as low as that of CWR. There is no doubt that the value of the tangential force depends to a large extent on the value of angle *β*, which was twice as large for CWR as for HWR. In both processes, the time required to produce one stud was about two seconds, with the reservation that, in the case of the cross-wedge method, it was necessary to add the time needed for the return of the tool and feeding the next billet into the mill. In the case of HWR, the forming path was far longer. Therefore, in order to compare these two processes in terms of energy, the amount of work needed to obtain one stud was determined. Energy for the analyzed processes was determined by integrating the field under the tangential force distribution for the CWR process and torque for the HWR process. The work required to produce one stud was 12.25 kJ for CWR and 8.89 kJ for HWR. It follows that in HWR, the energy demand was 27.5% lower than in CWR.

**Figure 12:**
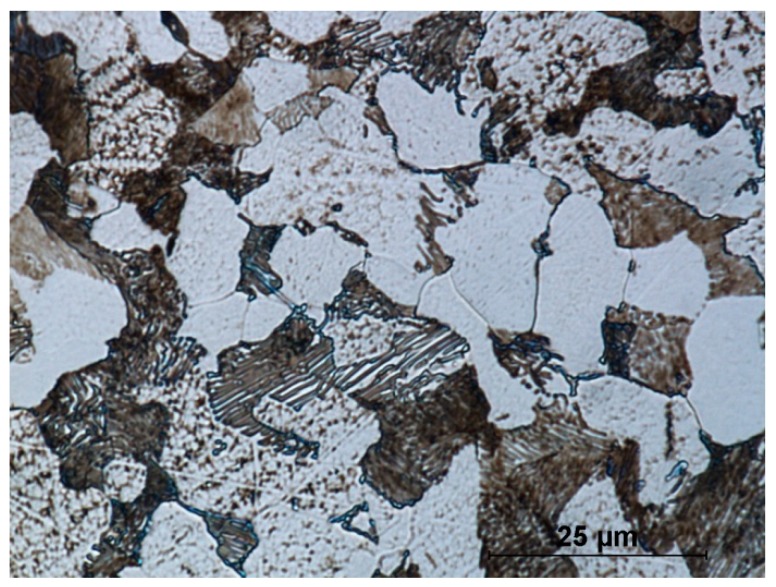
Microstructure of C45 steel in delivery condition.

Figure 12 shows the microstructure of the stock material. Steel in delivery condition has a ferrite–pearlite structure. Clearly visible is the lamellar structure of cementite and ferrite inside perlite grains. The steel is uniform throughout the cross-section of the rod. The ferrite and perlite grains are the same size. The mean grain size of the stock material is 17 μm.

**Figure 13:**
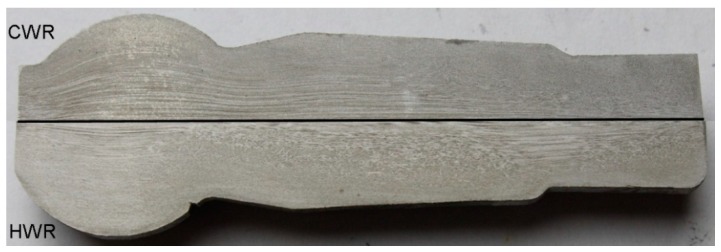
Material flow lines observed after etching in Jacewicz’s reagent (38 cm^3^ HCL + 12 cm^3^ H_2_SO_4_ (1.83 g/cm^3^) + 50 cm^3^ H_2_O).

Figure 13 presents a macroscopic view of the obtained ball pins with the material flow lines shown. In the case of CWR forging material, flow lines in the central part of the forging are located along its axis. As far as the surface layers of the forging are concerned, directional metal flow does not occur. In the case of HWR forging material, flow lines along the axis of the forging can be observed in a small area near the axis of the forging. In both cases, the presented lines of material flow are reflected in the distribution of effective strain (Figure 6).

**Figure 14:**
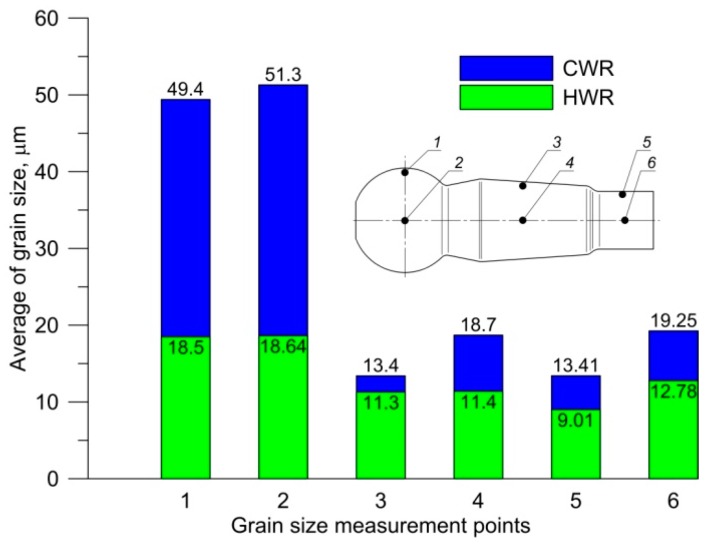
Average size of grain measures in six points.

**Figure 15:**
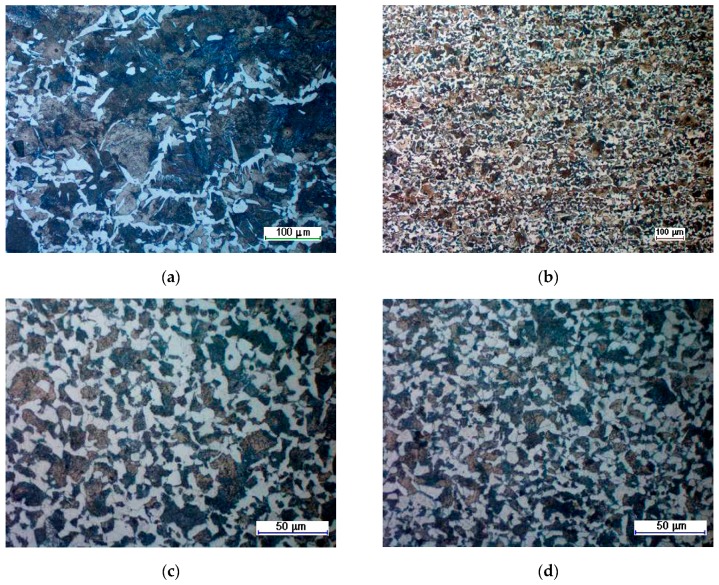
Exemplary photographs of the microstructure of the forgings manufactured using: (**a**) cross-wedge rolling (CWR) in point 2; (**b**) the helical-wedge rolling (HWR) method in point 2; (**c**) CWR in point 5; (**d**) HWR in point 5.

Figure 14 presents an average grain size of a ferritic–pearlitic structure, measured in six selected areas. The average grain size measurement was performed in accordance with the Jeffries planimetric grain size method [19]. In order to count the grains, they were arranged in circles, located in three spots in the microstructure display. ImageJ 1.52i software was used for creating the circles and grain counting. Based on the obtained results, it can be stated that the microstructure of the forging obtained in the HWR process was more fine-grain that the microstructure of the forgings obtained in the CWR process. In the case of the HWR process, the homogeneity of the structure was more significant than in the case of CWR. In the CWR, forging aggregations of big pearlite grains in fine-grain ferritic matrix (Figure 15a) are observed in the areas of insignificant strain (ball of the ball pin). In the remaining areas, the microstructure of the CWR forging was homogenous and fine-grained (Figure 15c).

## 4. Conclusions

This paper presents the results of numerical simulations and experimental investigations of two rolling processes. Cross-wedge rolling and helical-wedge rolling processes were compared using the example of rolling a C45 steel ball stud at 1100 °C. The results of the study lead to the following conclusions:

- HWR is a more efficient and less material-intensive technology than CWR.
- Separation of forgings is more stable during HWR and does not cause deformation of the forming parts.
- In HWR, the values of plastic deformation are greater, which may be the consequence of the workpiece being processed over a longer forming path.
- CWR is associated with higher values of the damage criterion.
- The maximum values of radial forces are similar for HWR and CWR.
- The tangential force in CWR is more than twice as large as the tangential force in HWR.
- The amount of energy consumed in rolling one ball stud is lower for the HWR method.
- The microstructure of the forgings rolled using the HWR method at 1100 °C is more fine-grained and homogenous than in the forgings manufactured with CWR.

## Figures and Tables

**Table 1:** Percentage of chemical elements for C45 steel determined by emission spectrometry.

Steel	C	Mn	Si	P	S	Cr	Mo	Ni	Al	Cu	Ti	Fe
C45	0.47	0.63	0.23	0.01	0.01	0.097	0.015	0.11	0.02	0.23	0.012	rest
